# Strongylus, Seu Eustrongylus Gigas, Ascaris Renalis

**Published:** 1882-04

**Authors:** Edward S. Breeder


### IV.—STRONGYLUS, SEU EUSTRONGYLUS GIGAS, ASCARIS RENALIS.

REPORTED BY EDWARD S. BREEDER, D.V.S.

The horse afflicted was a bay gelding, æt. 6. He suddenly became lame about one week before he died, while being driven to a single truck.

When first examined he was found to have a centre crack on the right posterior foot.

For three days he was treated by a surgeon, who then gave the animal up, as he was too sensitive to allow any local treatment unless placed under anæsthetics.

He was then sent to the Columbia Veterinary College Hospital in an ambulance.

Within a few days before he became lame he had rapidly emaciated, but prior to that had always been quite well and very serviceable.

The day following his admission to the hospital the house surgeon noticed that the urine passed by the animal was thick, slimy, and of a deep yellow color. It was then evident that the centre crack was not the only trouble, and it was certain he was also suffering great bodily pain. He rapidly became prostrated. Opiates were freely administered, but with no apparent relief, and he died that evening.

A *necropsy* was made the following day.

*Thoracic Cavity.*—The pericardial sac was congested, but the amount of fluid was about normal. The heart cavities contained large post-mortem clots, but firmer than those commonly met with. They were of a dirty yellow color and resembled an ante-mortem clot. These clots extended from the cavities of the heart well into the pulmonary artery and its branches, and also into the aorta. The one removed from the pulmonary artery was about two feet long and was composed of many shreds.

The heart was soft, pale and fatty. The walls of both ventricles were not more than half the normal thickness; of a pale, yellow color, as compared with the normal, and both were very fatty.

At one point of the endocardium there was positive evidence of endocarditis in the shape of an endocardial ulcer.

The lungs were quite free in their pleural sacs. Each sac contained some serous fluid, and the right a little clotted blood. Both lungs were œdematous and deeply congested. The base of the right lung presented a peculiar appearance, the substance being partly destroyed and resembling an open network of fibres, with a sponge-like appearance—resembling the angiomatous developments sometimes met with.

The lungs were also studded with numerous dentate calcareous masses, the centres of which contained a fluid substance.

*Abdominal Cavity.*—The peritoneal sac contained a large amount of blood and coagulum, also twenty-one round worms, varying in length from nine to twelve centimetres. They were of the lumbricord variety.

The alimentary tract was normal, with the exception of the stomach, which was a little softened.

The genito-urinary tract, up to the kidneys, appeared normal. Both glands were the seat of very interesting changes, presenting a very peculiar appearance. They were full of small round holes about one centimeter in diameter. These holes were very numerous, giving the kidneys a worm-eaten appearance. This change was supposed to be due to the passage of the round worms through the gland tissue for two reasons. First, the holes passed through the capsule, as well as the kidney tissue, and a second worm was found in one of the kidneys. Microscopic examination of the organs showed that the renal cells were slightly granular, but otherwise quite normal in appearance. Transverse sections of the openings gave them the appearance of pretty clean-cut holes at the expense of the kidney structure. Each canal was surrounded by a thin layer of inflammatory exudation; but the inflammatory action was limited to a very thin area, and evidently had not irritated the gland tissue to any marked degree.

The *liver* was nearly destroyed, little else remaining than a mass of cavernous tissue which was rapidly passing into a gangrenous condition. In its removal it was torn in various directions, and it was quite apparent that it had given way before or just after death, and it was probably from the liver that the peritoneal hæmorrhage came.

NEW YORK CITY, March, 1882.

[This case is one of the exceptional cases met with in medicine. The strongylus gigas is said to be common to man, horses, oxen and dogs. There is another form of parasite which is said to occur in the human kidney, which is not mentioned in Prof. Law's table of parasites common to various animals. Possibly the Tetrastoma renalis is the more common form when it occurs in the human kidney. This parasite, however, is not generally believed to inhabit the intestines, and undoubtedly this case is that properly entitled Strongylus gigas.—ED.]

